# Case Report: Androgen-secreting pediatric adrenocortical tumor of uncertain malignant potential presenting with virilization in a 19-month-old girl

**DOI:** 10.3389/fendo.2026.1899515

**Published:** 2026-07-23

**Authors:** Yirong Wang, Bo Sun, Xiangyue Lu, Tongmin Li, Mei Han, Hongbin Hou

**Affiliations:** 1 Department of Endocrinology/Pediatric Endocrinology, Genetics and Metabolism, Gansu Provincial Maternity and Child Care Hospital (Gansu Provincial Central Hospital), Lanzhou, China; 2 The First Clinical Medical College, Lanzhou University, Lanzhou, China

**Keywords:** adrenocortical tumor, pediatric endocrinology, peripheral precocious puberty, uncertain malignant potential, virilization, Wieneke score

## Abstract

**Background:**

Virilization in girls younger than 2 years should prompt careful evaluation for pathological androgen excess and should be interpreted as a practical clinical reminder rather than as a stand-alone diagnostic tool. Pediatric adrenocortical tumors (ACTs) are rare but are usually hormonally active, and their interpretation requires pediatric-specific clinicopathological criteria rather than direct application of adult adrenal carcinoma criteria.

**Case presentation:**

A 19-month-old girl presented with a 6-month history of progressive voice deepening, pubic hair growth, and clitoromegaly. Beard-like facial hair, acne, axillary hair, adult-type body odor, and vaginal bleeding were not reported. Basal gonadotropins were suppressed, gonadotropin-releasing hormone analogue stimulation showed a non-pubertal luteinizing hormone response, bone age was advanced, and dehydroepiandrosterone sulfate was markedly elevated (>1,000 μg/dL; above the assay upper reportable limit). Imaging identified an approximately 5-cm right adrenal mass. The patient underwent open surgical resection. Histopathology showed an ACT of uncertain malignant potential with a formally reported Wieneke score of 3, based on increased or borderline-high mitotic activity as interpreted in the pathology report, atypical mitotic figures, and capsular invasion. Immunohistochemistry showed steroidogenic marker expression, a missense-mutant p53 pattern, focal reticulin rarefaction, and a Ki-67 hotspot index of approximately 30%. Postoperative androgen levels declined markedly, and biochemical remission was maintained at 4 and 8 months.

**Discussion:**

Androgen-dominant virilization preceded and outweighed mild breast development, serving as a practical reminder to prioritize adrenal-focused assessment in very young girls with rapidly progressive virilization. Preoperative ultrasonography, contrast-enhanced computed tomography, and magnetic resonance imaging were performed; dedicated postoperative contrast-enhanced abdominal CT or MRI had not yet been obtained during the available follow-up period. Genetic testing was incomplete because the family declined germline testing after counseling; testing of the proband should be re-offered during follow-up, with cascade testing for first-degree relatives if a pathogenic germline variant is identified.

**Conclusion:**

Long-term surveillance should include endocrine assessment, pubertal monitoring, genetics re-counseling, and protocolized cross-sectional imaging, because early biochemical remission does not exclude recurrence or later secondary central precocious puberty.

## Introduction

Precocious puberty is broadly classified into central (gonadotropin-dependent) and peripheral (gonadotropin-independent) forms. Peripheral precocious puberty results from exposure to sex steroids despite a prepubertal hypothalamic-pituitary-gonadal axis. In girls, physiological puberty typically begins with thelarche; by contrast, early pubarche, clitoromegaly, acne, growth acceleration, and voice deepening are signs of androgen excess and should trigger directed evaluation rather than expectant management (1, 2).

Pediatric ACTs are rare, with an estimated annual incidence of 0.3–0.4 per million children, yet most are hormonally active and frequently present first to pediatric endocrinologists rather than surgeons (3–5). Their endocrine phenotype, molecular background, and prognostic determinants differ from those of adult adrenal cortical neoplasms, and pediatric-specific pathological interpretation is therefore required (3, 4, 6–8). Moreover, a substantial proportion of pediatric ACTs harbor germline TP53 alterations or chromosome 11p15 abnormalities, further distinguishing them from sporadic adult adrenal tumors (9–11).

Two previously reported virilizing pediatric ACTs provide context for the present case and help define its additional educational value, though they differ in several clinically relevant respects. Madi et al. described a 6-year-old girl whose symptoms began at approximately 5.5 years of age; she had a functional oncocytic adrenocortical tumor—a distinct histological subtype characterized by abundant eosinophilic cytoplasm and mitochondrial-rich cells, for which the Lin-Weiss-Bisceglia oncocytic-specific criteria are recommended over standard Weiss-type or Wieneke scoring (12). That patient developed central precocious puberty 3 months after resection. Goyal et al. described a 7-year-old boy with an 8-month history of progressive phallus enlargement and premature pubic hair, diagnosed with adrenocortical carcinoma using conventional adult-type Weiss criteria, who developed central precocious puberty 6 months after surgery (13). By comparison, our patient presented at a much younger age (symptom onset at approximately 13 months), was female with heterosexual virilization, had a non-oncocytic tumor assessed using the pediatric-specific Wieneke system (score of 3, uncertain malignant potential), and has shown no evidence of secondary central precocious puberty through 8 months of follow-up. These differences in age at symptom onset, gender and pubertal presentation, histological subtype and classification system, and postoperative course highlight the heterogeneity of pediatric ACTs and underscore the importance of pediatric-specific diagnostic frameworks.

The added value of the present report is therefore educational and integrative: it shows, in an infant younger than 2 years, how the chronological predominance of virilization, adrenal steroid localization, avoidance of biopsy, pediatric-specific histological classification, genetic counseling, and explicit acknowledgment of staging and surveillance gaps can be combined into a practical diagnostic and follow-up framework.

## Case description

A 19-month-old girl was admitted to the pediatric endocrinology service on September 19, 2025, after approximately 6 months of progressive voice deepening and pubic hair growth. The first symptoms therefore appeared at approximately 13 months of age. Her caregivers had also observed clitoromegaly. Beard-like facial hair, acne, axillary hair, adult-type body odor, and vaginal bleeding were not reported. The absence of beard-like facial hair or other overt hirsutism did not exclude androgen excess, because virilizing manifestations in very young children may vary according to age, duration of androgen exposure, androgen concentration, and regional hair-follicle sensitivity. Breast development had not clearly preceded the virilizing features. Exogenous exposure to sex steroids—including dietary supplements, topical hormonal preparations, and environmental sources—was denied. The patient was born at term by cesarean delivery with a birth weight of 3,400 g and a birth length of 51 cm. The available family history was reportedly unremarkable. According to the caregivers, there was no known family history of childhood cancers, adrenocortical tumors, sarcomas, early-onset breast cancer, brain tumors, leukemia, osteosarcoma, or other malignancies suggestive of Li-Fraumeni syndrome among first-degree relatives; however, a formally documented extended pedigree was not available.

On admission, height was 88 cm and weight was 14 kg, both above the 97th percentile for age and sex, consistent with growth acceleration. Blood pressure was 92/68 mmHg. Pubertal staging revealed Tanner B2 breasts, pubic hair Tanner PH2–3, and clitoromegaly. The sequence of pubertal signs was diagnostically informative: virilization was the earliest and most prominent manifestation, whereas breast development was mild and not reported as the initial sign.

Basal follicle-stimulating hormone (FSH) and luteinizing hormone (LH) were both <0.30 IU/L (prepubertal reference: FSH <1.0 IU/L; LH <0.3 IU/L). Triptorelin stimulation testing yielded LH values of 1.86, 3.64, 1.68, and 1.59 IU/L at 30, 60, 90, and 120 minutes, respectively; these responses were interpreted as non-pubertal. Bone age, assessed using the Greulich and Pyle atlas method, was advanced to approximately 2–3 years at a chronological age of approximately 19 months. Preoperative laboratory values are summarized in **Table 1**, together with typical prepubertal reference ranges provided for general orientation in a separate column. Key findings included DHEAS >1,000 μg/dL (reference for 1–2 years: <20 μg/dL), testosterone 5.95 ng/mL (prepubertal female reference interval: 0.029–0.481 ng/mL), and 17-hydroxyprogesterone 8.8 nmol/L (reference interval: 0–12 nmol/L). Morning cortisol was 5.98 μg/dL (reference interval: 6.2–18 μg/dL) and adrenocorticotropic hormone (ACTH) was 3.98 pg/mL (reference interval: 7.2–63.3 pg/mL).

**Table 1:** Endocrine profile before surgery and during postoperative follow-up.

Hormone	Reference interval	Preoperative (2025-09-19)	POD 18 (2025-10-17)	4-Month (2026-01-23)	8-Month (2026-05-10)
FSH (IU/L)	Prepubertal: <1.0	<0.30	1.3	2.7	2.1
LH (IU/L)	Prepubertal: <0.3	<0.30	<0.30	<0.30	<0.30
Estradiol (pg/mL)	Prepubertal female: <15	11.10	<5.00	<5.00	<5.00
Prolactin (ng/mL)	4.79–23.3	17.8	6.9	15.80	8.60
Progesterone (ng/mL)	Prepubertal: <0.5	1.15	0.10	0.140	0.120
17-OHP (nmol/L)	0–12	8.8	NA	NA	NA
Testosterone (ng/mL)	0.029–0.481	5.95	0.03	0.03	0.03
DHEAS (μg/dL)	1–2 years: <20	>1,000	0.22	1.09	1.18
Morning Cortisol (μg/dL)	6.2–18	5.98	7.35	7.25	12.20
ACTH (pg/mL)	7.2–63.3	3.98	30.31	35.60	39.90

POD, postoperative day; 17-OHP, 17-hydroxyprogesterone; NA, not available. Values are reported in the original local laboratory units. The preoperative DHEAS concentration exceeded the upper reportable limit of the local assay (>1,000 μg/dL). Reference ranges shown are derived from published literature for prepubertal girls where available, except for testosterone and 17-hydroxyprogesterone, for which the local laboratory’s stated reference ranges are shown. All reference ranges are provided for general orientation only, and assay- and age-specific reference intervals from the local laboratory should be confirmed and reported where available.

**Figure 1:**
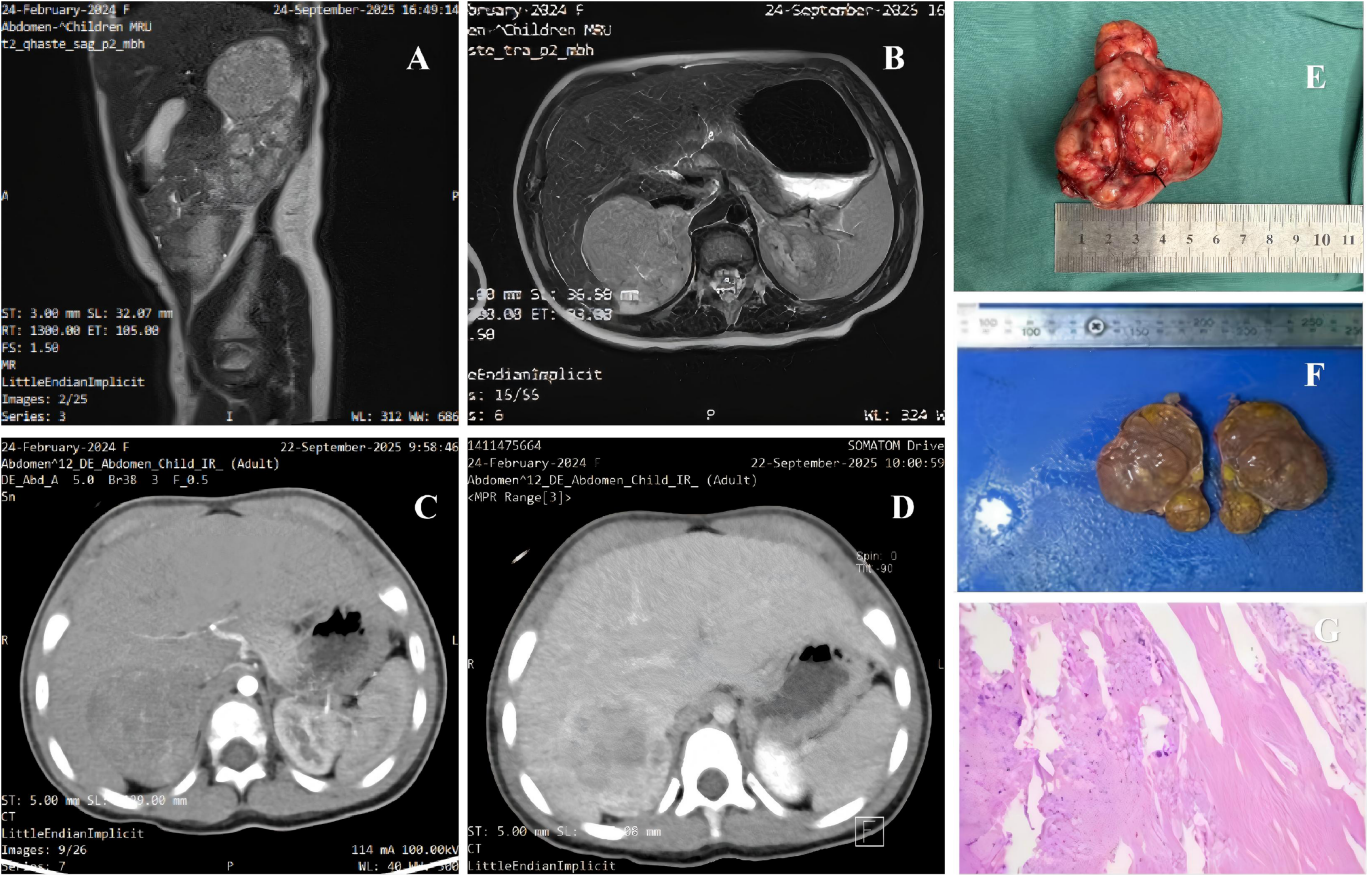
Preoperative imaging, gross specimen, and histopathological findings of the right adrenal tumor. **(A)** Sagittal MRI showing a well-circumscribed round mass in the right adrenal region with slightly hyperintense signal characteristics. **(B)** Axial MRI demonstrating an irregular right adrenal mass with heterogeneous internal signal intensity and mass effect on adjacent structures. **(C)** Contrast-enhanced CT showing a heterogeneous right adrenal lesion measuring approximately 5 cm with irregular enhancement. **(D)** Delayed-phase CT image demonstrating persistent enhancement with patchy low-density necrosis-like areas within the lesion. **(E)** Gross postoperative specimen showing a lobulated solid tumor with a relatively intact capsule. **(F)** Cut surface of the tumor showing a gray-yellow to brown-yellow solid appearance with nodular changes. **(G)** Histopathological examination (hematoxylin–eosin staining) showing diffuse sheets of tumor cells with cellular atypia and focal capsular invasion.

Pelvic ultrasonography revealed a small uterus with an indistinct body–cervix boundary and no dominant ovarian mass. Adrenal ultrasonography demonstrated a right adrenal hypoechoic mass measuring 58 × 54 × 48 mm. Contrast-enhanced computed tomography (CT) showed a right adrenal lesion measuring approximately 5.0 × 4.7 × 4.9 cm with an unenhanced attenuation of approximately 37 Hounsfield units, heterogeneous arterial phase enhancement, progressive venous phase enhancement, and patchy low-density areas on delayed imaging. Magnetic resonance imaging (MRI) revealed an irregular right adrenal mass measuring approximately 4.3 × 5.1 × 5.4 cm, isointense on T1-weighted imaging, slightly hyperintense on T2-weighted imaging, hyperintense on diffusion-weighted imaging, and hypointense on apparent diffusion coefficient mapping. Representative preoperative imaging and gross specimen photographs are shown in **Figure 1**.

Open surgical resection was performed on September 29, 2025, via a right subcostal incision approximately 8 cm in length. The tumor was located beneath the right hepatic lobe, above the right kidney, and posterior to the inferior vena cava (IVC). It appeared gray-white, irregular in shape, with an intact capsule and a relatively well-defined plane from adjacent structures, although a portion of the lesion was closely associated with the adrenal cortex. A prominent draining vessel communicating with the IVC territory was identified, ligated, and transfixed. The tumor measured approximately 6 × 5 × 4 cm intraoperatively. No obvious regional lymphadenopathy was observed, and no IVC thrombus was palpable. Estimated blood loss was minimal, and transfusion was not required. Retroperitoneal drainage was placed after meticulous hemostasis. The operative record documented no gross residual tumor; however, formal microscopic surgical margin status was not explicitly reported and is therefore treated as a limitation of this report.

**Figure 2:**
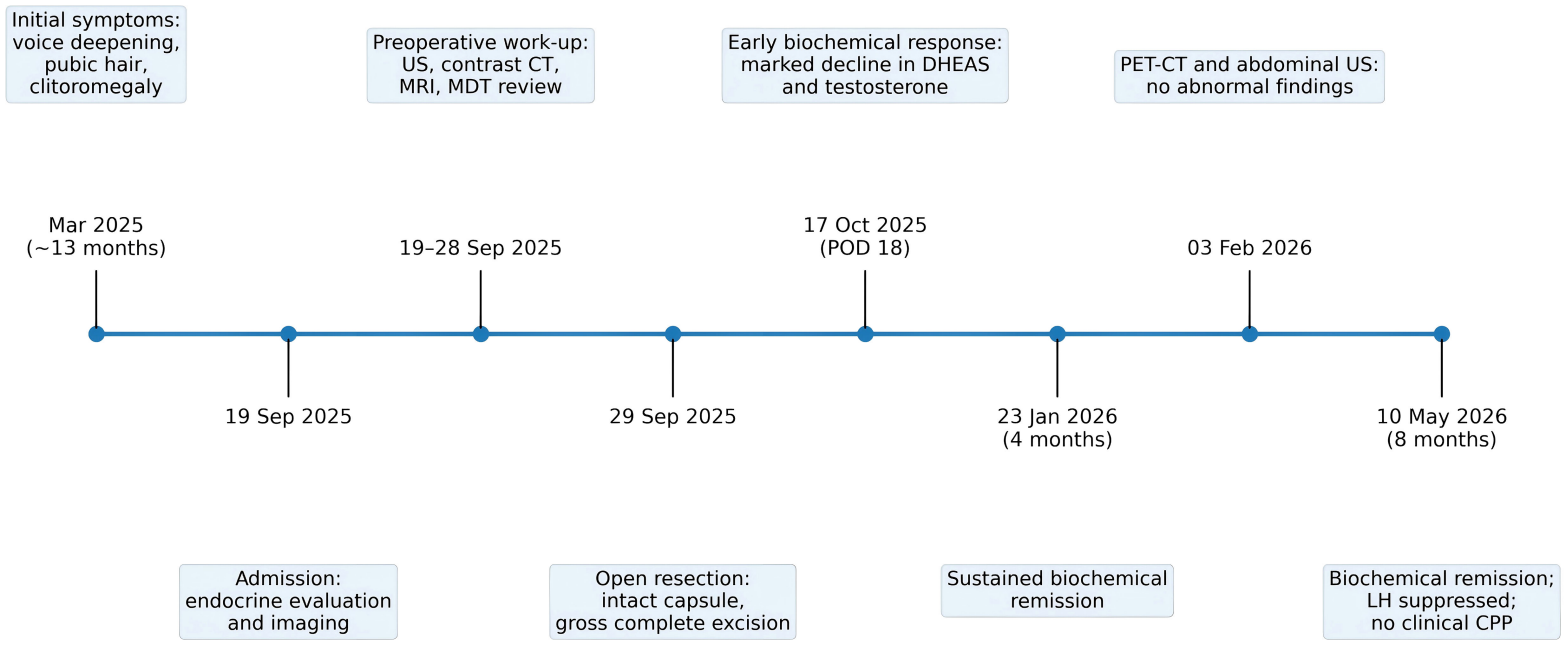
Graphical clinical timeline summarizing symptom onset, diagnostic evaluation, surgery, biochemical response, and postoperative follow-up. CPP, central precocious puberty; CT, computed tomography; DHEAS, dehydroepiandrosterone sulfate; LH, luteinizing hormone; MDT, multidisciplinary team; MRI, magnetic resonance imaging; PET-CT, positron emission tomography–computed tomography; POD, postoperative day; US, ultrasonography.

A graphical clinical timeline of key events is presented in **Figure 2**.

## Diagnostic assessment and therapeutic intervention

### Diagnostic assessment

No adrenal biopsy was performed before surgery. In a child with biochemical and radiological suspicion of a resectable functional adrenocortical tumor, biopsy is generally not recommended because it may risk tumor rupture or seeding, does not reliably establish malignant potential, and should not delay complete surgical excision. The combination of suppressed basal gonadotropins, a non-pubertal GnRH analogue response, and markedly elevated DHEAS (>1,000 μg/dL; reference for 1–2 years: <20 μg/dL) localized the androgen excess to the adrenal cortex rather than to the gonads or the central hypothalamic-pituitary-gonadal axis (1). The differential diagnosis included congenital adrenal hyperplasia (CAH), exogenous androgen exposure, premature adrenarche, and androgen-secreting adrenal or gonadal tumors. Classic CAH was considered unlikely because 17-hydroxyprogesterone was not elevated according to the local laboratory interpretation, imaging demonstrated a unilateral adrenal mass, gonadotropins were suppressed, and the androgen excess improved after tumor removal. Premature adrenarche was inconsistent with the very young age, rapid progression, markedly elevated DHEAS, clitoromegaly, and voice deepening. No exogenous androgen exposure was identified. Preoperative androstenedione and 11-deoxycortisol were not measured, which limited full characterization of the adrenal steroid profile and restricted assessment of possible cortisol co-secretion. Morning cortisol was slightly below the local reference interval, whereas ACTH was low; however, without a complete steroid profile and dynamic cortisol assessment, autonomous cortisol secretion could not be confirmed or excluded.

Multimodal imaging—including ultrasonography, contrast-enhanced CT, and MRI—consistently demonstrated a large, heterogeneous right adrenal mass with imaging features concerning for malignancy. The endocrine and imaging findings together raised a high index of suspicion for a functional adrenal cortical neoplasm with malignant potential. Formal pediatric tumor staging was limited because dedicated chest CT staging was not performed before surgery; therefore, the absence of overt metastatic disease should be interpreted cautiously.

### Histopathology

Histopathological examination classified the lesion as an ACT of uncertain malignant potential. The exact mitotic count reported by pathology was 15 mitoses per 20 high-power fields. This value lies at the threshold used in Wieneke-based assessment; although the formal pathology report categorized mitotic activity as increased and included it in the score, we report the count transparently and interpret this adverse feature cautiously within the complete Wieneke framework rather than as an isolated diagnostic criterion. The specimen weighed <400 g and measured approximately 6.5 × 3.5 × 4 cm. The pathology report documented increased or borderline-high mitotic activity as described above, atypical mitotic figures, and capsular invasion by tumor tissue. No obvious venous, sinusoidal, or vascular invasion was identified, and tumor necrosis or extra-adrenal extension was not documented.

Immunohistochemistry showed the following profile: SF-1 positive, Melan-A positive, inhibin-α positive, synaptophysin positive, chromogranin A negative, β-catenin (cytoplasmic) positive, p53 missense-mutant pattern, RB protein positive, CDK4 negative, calretinin negative, and scattered cytokeratin positivity. The Ki-67 hotspot index was approximately 30%. Reticulin staining demonstrated focal reticulin fiber rarefaction.

The Wieneke scoring system was applied (14). To avoid overinterpretation, the score was not treated as a binary malignant-versus-benign label but as a pediatric risk-stratification framework. Under this system, nine histopathologic parameters are evaluated: tumor weight >400 g, tumor size >10.5 cm, extension into periadrenal soft tissue or adjacent organs, venous invasion, capsular invasion, tumor necrosis, increased mitotic rate, atypical mitotic figures, and sinusoidal invasion. The positive adverse features documented in the formal pathology interpretation were increased or borderline-high mitotic activity, atypical mitotic figures, and capsular invasion, yielding a total Wieneke score of 3. Given the borderline mitotic count, the overall classification was presented conservatively as uncertain malignant potential rather than unequivocal carcinoma. The tumor did not meet the weight or size thresholds, and definite venous, sinusoidal, or vascular invasion was not identified. A diagnosis of ACT of uncertain malignant potential is consistent with this score (14, 15).

### Therapeutic intervention

Complete surgical excision without tumor rupture is the cornerstone of potentially curative treatment for localized pediatric ACTs (4–6, 16). In this patient, the open subcostal approach was appropriate given the tumor’s proximity to the liver, right kidney, and IVC, and the operative findings confirmed capsule integrity, adequate vascular control, absence of gross nodal disease, and a tumor-free IVC. No preoperative biopsy was performed, and no adjuvant chemotherapy or radiotherapy was administered, consistent with current recommendations for completely resected pediatric ACTs of uncertain malignant potential in the absence of clinically evident metastatic disease (4–6, 16).

### Follow-up and outcomes

Postoperative testosterone and DHEAS declined markedly by postoperative day 18 and remained low at the 4- and 8-month visits, supporting early biochemical remission after tumor resection. At 8 months, basal LH remained suppressed and there was no clinical evidence of progressive thelarche or secondary central precocious puberty. Postoperative positron emission tomography–computed tomography (PET-CT) and abdominal ultrasonography showed no abnormal findings, but these studies were not considered a substitute for protocolized postoperative contrast-enhanced abdominal CT or MRI surveillance. Preoperative MRI and contrast-enhanced CT had been performed; dedicated postoperative contrast-enhanced abdominal CT or MRI had not yet been obtained during the available follow-up period. Dedicated chest CT staging was not available, which limits certainty about baseline thoracic metastatic assessment.

## Discussion

This case demonstrates the clinical value of carefully documenting the sequence of pubertal signs in very young girls. We do not propose this sequence as a new diagnostic tool; rather, we emphasize it as a practical reminder that can prevent premature classification as central precocious puberty and can prompt timely adrenal-focused evaluation. Central precocious puberty is estrogen-led and typically begins with thelarche; by contrast, pubic hair growth, clitoromegaly, rapid growth, and voice deepening point to androgen excess from an adrenal or gonadal source (1, 2). In this patient, virilization began at approximately 13 months of age and clearly preceded estrogen-dominant development. DHEAS, produced predominantly by the adrenal cortex, was the most informative biochemical finding: markedly elevated DHEAS (>1,000 μg/dL; reference for 1–2 years: <20 μg/dL), suppressed gonadotropins, a non-pubertal GnRH analogue response, and a unilateral adrenal mass together confirmed adrenal-origin peripheral precocious puberty. The postoperative decline in adrenal androgens further confirmed that the tumor was functional.

### Histological pattern and classification

The present tumor was classified as an ACT of uncertain malignant potential using the pediatric-specific Wieneke scoring system, with a score of 3 according to the formal pathology interpretation of increased or borderline-high mitotic activity, atypical mitotic figures, and capsular invasion (14). The tumor did not meet the adverse weight (>400 g) or size (>10.5 cm) thresholds, and venous invasion, sinusoidal invasion, necrosis, and extra-adrenal extension were not documented. A Wieneke score of 3 indicates indeterminate malignant potential rather than unequivocal carcinoma. In a systematic review of 128 pediatric ACT patients, the Wieneke/AFIP criteria had 92% sensitivity and 77% specificity for malignant behavior, with approximately 15% of histologically malignant tumors nonetheless following a benign clinical course (17); in a French national cohort of 95 pediatric ACTs, 5-year progression-free survival was 82%, with no recurrences when Ki-67 was <15% (18). In the present case, the elevated Ki-67 hotspot index (approximately 30%) and missense-mutant p53 pattern supported the need for careful surveillance but did not alone establish germline disease or metastatic behavior.

### Genetic testing

Pediatric ACTs are associated with cancer-predisposition syndromes, particularly germline TP53 alterations (Li-Fraumeni syndrome), and chromosome 11p15 abnormalities may also be relevant (9–11). The missense-mutant p53 immunostaining pattern in this case raised concern for TP53 pathway involvement; however, p53 immunohistochemistry cannot determine whether the underlying alteration, if present, is somatic or germline. Therefore, the staining pattern supported the need for genetic counseling but could not confirm Li-Fraumeni syndrome. Germline testing was recommended but remained incomplete because the family declined testing after counseling. This decision should be revisited during follow-up; if a pathogenic germline TP53 variant is identified in the proband, first-degree relatives would have a 50% risk of carrying the same variant and should be offered cascade testing and syndrome-specific surveillance, given the autosomal dominant inheritance of Li-Fraumeni syndrome. The family’s anxiety regarding genetic testing, as expressed during counseling, highlights the need for continued engagement and re-counseling at subsequent follow-up visits.

### Postoperative surveillance and limitations

Early biochemical remission is reassuring but does not exclude recurrence or secondary central precocious puberty, particularly because the available staging and surveillance were incomplete. Prolonged exposure to peripheral sex steroids can activate the hypothalamic-pituitary-gonadal axis after tumor removal, as reported in the two prior pediatric cases discussed above (12, 13). Continued follow-up should include adrenal androgen profiling, growth velocity, pubertal staging, bone age, blood pressure, and repeat GnRH stimulation testing if progressive thelarche or growth acceleration develops. Periodic abdominal contrast-enhanced CT or MRI should be incorporated into postoperative surveillance, with chest imaging considered according to the treating team’s protocol. The limitations of this report should be considered when interpreting the case. The absence of preoperative androstenedione and 11-deoxycortisol measurements limits characterization of the steroid secretory pattern and evaluation of possible cortisol co-secretion. The lack of formal pediatric staging and dedicated chest CT limits certainty regarding baseline metastatic evaluation. The absence of dedicated postoperative contrast-enhanced abdominal CT or MRI during the available surveillance period limits radiological assessment of early local recurrence, although preoperative MRI and contrast-enhanced CT were performed. Unavailable microscopic margin status limits certainty regarding histological clearance. Incomplete germline testing prevents confirmation or exclusion of a hereditary cancer-predisposition syndrome. Finally, the short follow-up period limits prognostic inference. Therefore, this case should be interpreted primarily as an educational report illustrating early clinical recognition, endocrine localization, pediatric-specific pathological interpretation, genetic counseling, and transparent surveillance planning in a very young child with virilizing ACT, rather than as evidence supporting a new diagnostic tool or a generalizable prognostic conclusion.

## Conclusion

When virilization precedes estrogen-dominant development in a young girl, clinicians should use this pattern as a practical reminder to focus promptly on adrenal sources of androgen excess rather than as a stand-alone diagnostic rule. Complete surgical resection, pediatric-specific histological classification using the Wieneke system, genetic counseling with longitudinal re-engagement, and long-term clinical, biochemical, and imaging surveillance remain essential even when early postoperative biochemical remission is achieved.

## Patient perspective

The patient’s guardians—her mother and paternal grandmother, both serving as primary caregivers—reported that the progressive voice deepening, pubic hair growth, and clitoromegaly caused substantial distress and drove them to seek medical attention at multiple healthcare facilities before arriving at our center. In the local cultural context, the word ‘precocious puberty’ (性早熟, xing zao shu) carries significant stigma, and the family initially feared that the diagnosis would label their child as developmentally abnormal. The grandmother expressed particular guilt, wondering whether dietary or environmental factors during the child’s early life could have contributed. Following detailed explanation of the tumor-driven nature of the condition, the family experienced considerable relief upon seeing the marked biochemical improvement after surgery. They acknowledged their understanding of the importance of long-term surveillance, although they expressed ongoing anxiety about the possibility of tumor recurrence—anxiety that is compounded by their decision to decline germline genetic testing. They have agreed to continue structured follow-up and consented to the publication of this case, expressing the hope that sharing their experience would help other families recognize similar signs earlier and seek prompt specialist evaluation.

## Data Availability

The raw data supporting the conclusions of this article will be made available by the authors, without undue reservation.
